# Role of Osteocytes in Myeloma Bone Disease: Anti-sclerostin Antibody as New Therapeutic Strategy

**DOI:** 10.3389/fimmu.2018.02467

**Published:** 2018-10-24

**Authors:** Denise Toscani, Marina Bolzoni, Marzia Ferretti, Carla Palumbo, Nicola Giuliani

**Affiliations:** ^1^Department Medicine and Surgery, University of Parma, Parma, Italy; ^2^Department of Biomedical, Metabolic and Neural Sciences, Human Morphology Section, University of Modena and Reggio Emilia, Modena, Italy

**Keywords:** osteocytes, multiple myeloma, bone disease, sclerostin, immunotherapy

## Abstract

Osteocytes are terminally differentiated cells of the osteoblast lineage. They are involved in the regulation of bone remodeling by increasing osteoclast formation or decreasing bone formation by the secretion of the osteoblast inhibitor sclerostin. Monoclonal antibody anti-sclerostin, Romosozumab, has been developed and tested in clinical trials in patients with osteoporosis. In the last years, the role of osteocytes in the development of osteolytic bone lesions that occurs in multiple myeloma, have been underlined. Myeloma cells increase osteocyte death through the up-regulation of both apoptosis and autophagy that, in turn, triggers osteoclast formation, and activity. When compared to healthy controls, myeloma patients with bone disease have higher osteocyte cell death, but the treatment with proteasome inhibitor bortezomib has been shown to maintain osteocyte viability. In preclinical mouse models of multiple myeloma, treatment with blocking anti-sclerostin antibody increased osteoblast numbers and bone formation rate reducing osteolytic bone lesions. Moreover, the combination of anti-sclerostin antibody and the osteoclast inhibitor zoledronic acid increased bone mass and fracture resistance synergistically. However, anti-sclerostin antibody did not affect tumor burden *in vivo* or the efficacy of anti-myeloma drugs *in vitro*. Nevertheless, the combination therapy of anti-sclerostin antibody and the proteasome inhibitor carfilzomib, displayed potent anti-myeloma activity as well as positive effects on bone disease *in vivo*. In conclusion, all these data suggest that osteocytes are involved in myeloma bone disease and may be considered a novel target for the use of antibody-mediated anti-sclerostin therapy also in multiple myeloma patients.

## Introduction

Multiple Myeloma (MM) is characterized by uncoupling bone resorption and osteoblast (OB) formation resulting in severe bone formation inhibition leading to osteolytic bone lesions (1). Currently, only osteoclast (OCL) inhibitors such as bisphosphonates (BPs) and the monoclonal antibody anti- receptor activator of nuclear factor-κB ligand (RANKL) denosumab are FDA-approved for the treatment of MM bone disease. To date, studies investigating the bone anabolic effects of anti-MM drugs demonstrated that proteasome inhibitors stimulate osteogenic differentiation of human mesenchymal stromal cells and also improve the viability of osteocytes reducing apoptosis and autophagic cell death both *in vitro* and *in vivo* (2). Nevertheless, studies investigating new therapeutic targets and approaches that improve bone formation are strongly encouraged.

In recent years, there has been increasing interest in elucidating the role of osteocytes in MM bone disease and in developing new therapeutic strategy that target osteocyte functions. It is a widely accepted notion that osteocytes are involved in the regulation of physiological bone remodeling through the release of molecules that affect OCL and OB function. Moreover, recent studies demonstrated that MM cells induced apoptosis and autophagic cell death in osteocytes contributing to the increased activity of OCLs (2, 3).

Sclerostin (Scl) is a potent Wnt/β-catenin inhibitor secreted by mature osteocytes that control bone formation and resorption (4). Moreover, it has been demonstrated that MM cells increased Scl expression in osteocytes in MM murine models (5, 6) and its levels have been found elevated in MM patients in correlation with abnormal bone remodeling (7).

Indeed, the use of anti-Scl antibody (Scl-Ab) has been explored in experimental animal models of bone disorders demonstrating its efficacy in increasing bone formation and decreasing bone resorption (8, 9). In the clinical setting, the Scl-Abs romosozumab and blosozumab have been efficaciously tested in osteoporotic patients demonstrating potent activity in stimulating bone formation and reducing bone resorption (10, 11). While some research has been carried out on the feasibility of Scl-Ab therapy in MM mouse model, no clinical studies have been yet conducted among MM patients. In this perspective, the notion that Scl-Ab does not affect the activity of currently available anti-MM drugs (8) encourages the use of a combined therapy to treat skeletal disease and tumor progression.

The purpose of this review is to provide an overview of the role of osteocytes in MM bone disease describing the numerous improvements that have been made in this field. We first describe the osteocyte role in physiological bone remodeling as well as the importance of Scl in modulating their activity and functions. Moreover, we discuss the main mechanisms underlie the involvement of osteocytes in MM bone disease and the preclinical use of an immunotherapeutic approach based on Scl-Ab for improving bone disease in patients with MM.

## Osteocytes and bone remodeling

Osteocytes are cells belonging to the osteogenic lineage embedded in the bone matrix within the lacuno-canalicular cavities. They are derived from the original rounded OBs through conspicuous morphological and ultrastructural changes, such as reduction in size, in parallel with the formation and elongation of the cytoplasmic processes (12, 13). Osteocytes create an extensive network throughout the skeleton, by means of multiple dendrite-like processes, joining with the other bone cells (OBs/bone lining cells and stromal cells); this functional syncytium, based on interaction through intercellular junctions, is extended from the inner bone to the vascular endothelia (14–16). The bone cells' activity is involved in all bone processes, i.e., bone growth, bone modeling and bone remodeling. Bone remodeling induces bone turnover throughout life, i.e., the continuous skeletal “destruction” and “reconstruction,” in a dynamic manner, driven by the activity of osteoclastic and osteogenic cell lineages, thus allowing bone adaptation to both mechanical and metabolic requirements. This process also occurs in repairing skeletal damage, preventing accumulation of brittle hyper-mineralized bone, and maintaining mineral homeostasis by liberating stores of calcium and phosphorus (17). The activities of OCLs and OBs must be strictly regulated to ensure that bone homeostasis is maintained. Osteocytes are considered the key regulators to maintain this balance (18). Recently, signaling pathways by which the osteocyte exerts control over the other bone cells and also the potential ways in which these pathways may be exploited therapeutically have been investigated (19–29). In physiological conditions, the bone remodeling should occur when required. During targeted remodeling, which is the removal of a specific area of old or damaged bone, the initiating signal originates from the osteocytes that use their dendritic network to communicate to other cells (25, 30–33). On the other hand, it has been reported that the osteocyte damage, induced for example through the disruption of bone matrix canaliculi, may lead to release of paracrine factors that increase local angiogenesis and recruitment of OCL and OB precursors (21, 33–35). Other authors have suggested that another possible triggering event of the bone remodeling cycle is osteocyte apoptosis, as the increase of RANKL expression occurs concurrently, thus enhancing the osteoclastogenesis (36–38).

Osteocytes are mechanosensors (39–44) and capable of modulating OCLs and OBs that, together with the capillary blood supply, form the Basic Multicellular Unit (BMU), which is constantly replenished to perform the appropriate bone remodeling (17). To explain the remodeling activation due to mechanical requirements, Palumbo and coworkers (30), proposed a sequence of phases, through which osteocytes coordinate OCL and OB recruitment only when the micro-deformations induced by loading exceed the physiological range (i.e., fall above and below the lower and upper setpoint values, respectively) in mineralized matrix. The osteogenic cell system is organized in the Bone Basic Cellular System-BBCS (16), the functional syncytium formed by osteocytes, bone lining cells and stromal cells. The bone remodeling process is characterized by distinct phases. Under the above conditions, osteocytes drive steady ionic currents (45) outside the bone matrix to maintain the steady state. During unloading or when sensitivity to strain is altered by hormones, such as parathyroid hormone (PTH), estrogens etc, osteocytes stop producing a steady resting state ionic current and the bone lining cells, stromal cells and above all the osteocytes themselves (sensitive to loading changes) produce RANKL, as recently confirmed (27, 46–48) (1st Phase-Resorption). During the progression of erosive activity, the only cells which can inhibit OCLs are the surviving overstrained osteocytes that arrest OCL erosion when the local upper setpoint is exceeded. In this regard, it has been shown that an unexpected high number (about 60%) of osteocytes survive the end of OCL disruption (30). After this, the successive 2nd Phase-Reversion begins and the cells of the reversal phase (probably of stromal-fibroblast origin) differentiate into OBs. The exact signals that couple bone resorption to subsequent bone formation are not yet fully understood. Various authors believe that the cells of the reversal phase could be involved in sending or receiving these signals (22, 49, 50). It has also been postulated that OCLs may be the source of coupling factors, either secreting cytokines or via regulatory receptors and their membrane bound ligands (51). Other signaling pathways may include matrix derived factors such as bone morphogenic protein (BMP)-2), transforming growth factor β and insulin-like growth factor (19, 26). In the last 3rd Phase-Deposition, bone is progressively rebuilt. When the local strains fall again within the physiological range, the osteocytes in the newly-laid-down bone matrix restore the steady ionic current returning the bone to the resting state, therefore halting OB activity.

Osteocytes play a key role in remodeling modulation via secretion of antagonists of the Wnt signaling pathway, such as Scl (18). Scl, encoded by the gene SOST is secreted by osteocytes and negatively regulates Wnt signaling by binding the co-receptors low-density lipoprotein receptor-related protein (LRP5/6). During the new resting phase, osteocyte expression of the Wnt inhibitors SOST, and DKK-1/2 prevents further bone formation in the quiescent bone, (52, 53). Thus, during the bone remodeling cycle, Scl osteocyte expression declines leading to an OB-mediated new bone formation after bone resorption. Finally, newly formed osteocytes become entombed within the bone matrix and re-express SOST, resulting in cessation of bone formation.

## Role of sclerostin in the regulation of bone remodeling

Various molecular mechanisms, underlying the osteocyte's regulatory role in response to skeletal and mineral homeostasis, have been reported. As widely described by Sapir-Koren and Livshits (4), three categories of molecules are involved: (i) Scl, due to SOST promoter hypomethylation (54); (ii) the group of “mineralization-related genes,” involved in regulating mineralization and phosphate metabolism: dentin matrix protein 1 (DMP1), matrix extracellular phosphor glycoprotein (MEPE), and fibroblast growth factor 23 (FGF23) (18, 55, 56); (iii) proteins encoded by RANKL and OPG genes. Scl is currently considered the major mediator of the molecular osteocyte mechanisms involved in the process of adaptive bone responses. It is a 22-kDa glycoprotein produced by the SOST gene and displays both autocrine and paracrine effects. The SOST gene is mainly expressed in bone cells, although it is also expressed during fetal development in several tissues including cartilage, bone marrow (BM), pancreas, heart, aorta, liver, and kidney. However, postnatal expression of Scl is mostly limited to osteocytes, chondrocytes and cementocytes (57). In the mature skeleton, Scl is mainly synthesized by differentiated mature osteocytes entrapped within the mineralized matrix, while immature osteocytes, embedded in osteoid, bone lining cells and OBs, express very low levels of Scl.

Scl has provided a pivotal step in the knowledge of bone remodeling regulation. This central role is achieved through interplay between two opposing mechanisms: (1) unloading-induced high Scl levels, which simultaneously antagonize canonical Wnt in osteocytes and OBs and promote noncanonical Wnt and/or other pathways in osteocytes and OCLs (20, 58, 59); (2) mechanical loading-induced low Scl levels, that activates Wnt-canonical signaling and bone formation.

**Figure 1:**
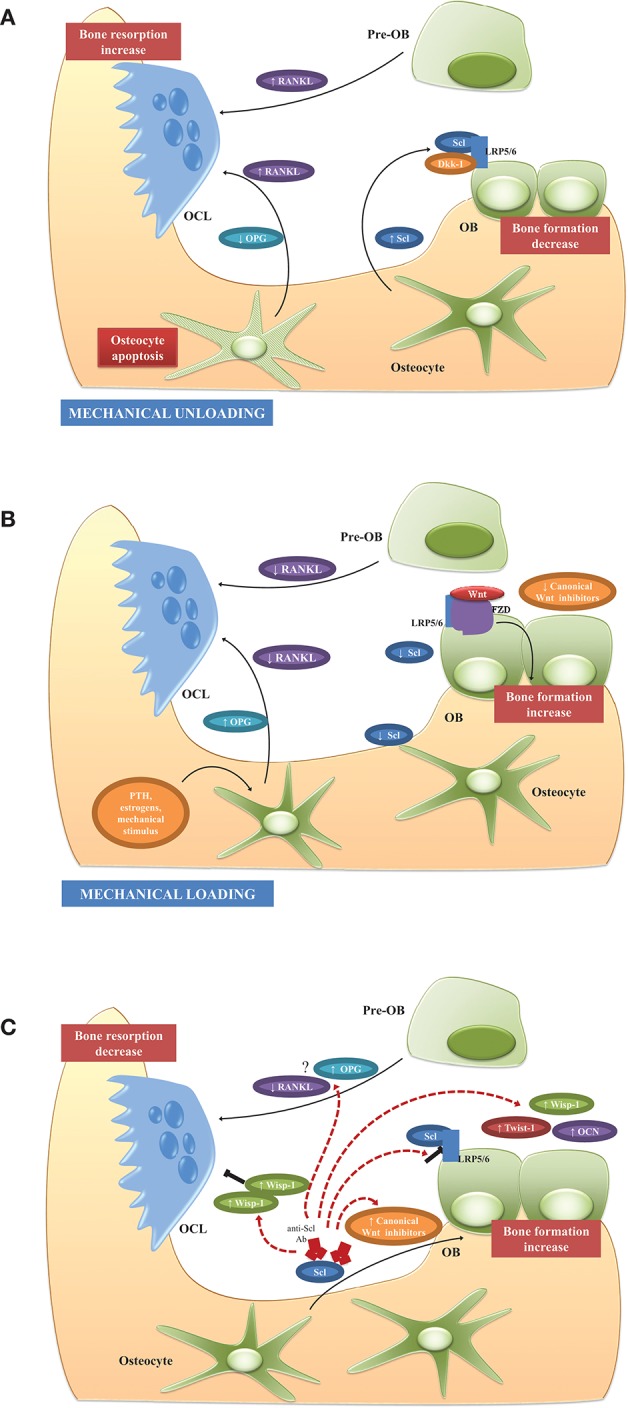
Physiological role of osteocytes and Scl and potential mechanism of action of Scl-Ab in BM microenvironment. **(A)** Unloading conditions induce the production of high Scl levels which, in turn, promote the production of RANKL and decrease OPG with consequent increased RANKL/OPG ratio, osteoclastogenesis, and enhanced bone resorption. Another possible triggering event of RANKL release in BM microenvironment is the osteocyte apoptosis. Simultaneously, high Scl inhibits Wnt signaling and osteoblast formation. **(B)** Mechanical loading and other factors, such as PTH and estrogens, suppress Scl expression with the consequent induction of Wnt signaling and enhanced bone formation. The production of high levels of OPG and the reduction of RANKL production lead to the suppression of resorption-associated activities. **(C)** Scl inhibition stimulates bone formation and reduces bone resorption by different mechanisms. Firstly, by blocking the binding between Scl and LRP5/6, Scl-Ab activates a set of Wnt target genes associated with bone formation and resorption (*Wisp* and *Twist*) and increased expression of extracellular matrix proteins, such as osteocalcin. The increased of Twist, an inhibitor of bone formation, limits the early response to Scl inhibition, whereas Wisp, a negative bone resorption, sustains the anti-osteoclastogenic activity. The feedback mechanisms following Scl inhibition, is associated with increased levels of Wnt antagonist to attenuate the bone-forming response and prevent excessive bone accrual. Although the anti-resorptive activity is demonstrated in animal studies and in clinical trials, the regulation of osteoclastogenic factors, such as RANKL and OPG, is not clearly and need to be elucidated in further studies. See text for details.

Thus, adaptive bone remodeling occurring in different bone compartments is driven by altered Scl levels, which regulate the expression of the other osteocyte-specific proteins, such as RANKL, its decoy receptor osteoprotegerin (OPG), and proteins encoded by “mineralization-related genes” (DMP1, PHEX, and probably FGF23). For example, under specific condition, Scl regulates differential RANKL, and OPG production, and creates a dynamic RANKL/OPG ratio (60–62), leading to either bone formation or resorption. It also controls the expression of PHEX, DMP1, and most likely FGF23 (55), leading to either bone matrix mineralization or its inhibition. Such opposite up- or down-regulation of the remodeling phases allows osteocytes (i.e., the cells always present in bone tissue) to function as “the orchestrators” of OCLs and OBs (i.e., the transient operating cells) ensuring the transition from bone resorption to bone formation. The physiological role of osteocytes and Scl in unloading and loading conditions is summarized in Figures 1A,B.

The inhibition of Scl could represent a promising strategy to target bone remodeling and has been investigated since 2009 in mouse and rat bone density disorder models (osteoporosis, rheumatoid arthritis, genetic disorders). In these models the use of Scl-Ab significantly increased bone mineral density (BMD), bone mass and strength and also OB surface while decreasing OCL surface (63, 64). Scl-Ab mechanism of action has been the focus of different studies. Specifically, in nonhuman primate and rat models, the short-term use of anti-Scl therapy improved and prolonged the bone formation by activating bone lining cells, while simultaneously reducing bone resorption (65, 66). In cynos, single dose of Scl-Ab, mimicking intermittent Scl inhibition, induced a rapid increase in serum procollagen type 1 amino-terminal propeptide (P1NP) and osteocalcin which returned to baseline as soon as the antibody was cleared from circulation (65). No increase in the serum levels of bone resorption marker C-telopeptide (CTX) was found in the serum levels, suggesting the anabolic effect of single and short treatment with Scl-Ab.

Interestingly, longer-term treatment resulted in a robust and transient increase in bone formation during the early phase of treatment followed by a progressive reduction. On the contrary, the anti-resorptive effects remained detectable throughout the whole period (67).

Expression analysis performed by microarray and TaqMan analysis on isolated OBs, bone-lining cells, and osteocytes isolated from both short-term and long-term Scl-Ab treated ovariectomized rats revealed the mechanisms underlying the bone response to Scl inhibition. Short-term treatment resulted in upregulated expression of canonical Wnt target genes: *Wisp1*, a negative regulator of bone resorption, and *Twist1* an inhibitor of bone formation. In the same conditions, an increased expression of all three osteogenic cell types of extracellular matrix and mineralization genes, such as *Bglap*, has also been observed within the first week of treatment (67, 68). Probably, the upregulation of *Twist* may limit the stimulatory response following Scl-Ab treatment. The progressive upregulation of matrix genes in lining cells supports the notion that Scl-Ab therapy differentiates lining cells into matrix-producing OBs on the quiescent surface without prior bone resorption (model-based bone formation) (68).

Interestingly, during extended treatment, at the time of peak bone formation rate, there was a decrease in the number of osteoprogenitor cells with a concomitant change in the global gene expression of osteocytes. In particular, *Twist1* returned to baseline levels while *Wisp1* remained increased suggesting a switch from anabolic to anti-anabolic expression profile in response to longer Scl-Ab treatment (67). The anti-resorptive activity of long-term treatment seemed to be accompanied by a reduction of *Csf1, a* gene encoding OCL regulator Macrophage Colony Stimulating Factor 1 (MCSF1), and an increased *Opg* expression (67). Further studies are needed to clarify how Scl-Ab modulates bone resorption since some authors reported the lack of modulation of RANKL, OPG, and other regulators of osteoclastogenesis during Scl-Ab treatment (68, 69).

Further pathways that inhibit canonical Wnt signaling such as Hippo, noncanonical Wnt (e.g., Wnat5b) and transforming growth factor (TGF)-β are significantly modulated by long-term treatment. These changes are likely driven by (i) increased p53, (ii) decreased c-Myc, and (iii) induction of Wnt inhibitors production dickkopf (Dkk)-1 and Scl, resulting in a self-regulated inhibition of bone formation to prevent excessive bone accrual (67, 70). The main effects on the BM microenvironment during treatment with Scl-Ab are illustrated in Figure 1C.

## Clinical studies with Scl-Ab in skeletal disease

Given the numerous findings regarding the involvement of Scl in bone remodeling and bone disease, humanized Scl-Abs antibodies have been developed.

Romosozumab (AMG 785; Amgen, Thousand Oaks, CA, USA, and UCB, Belgium) is a humanized monoclonal IgG2 antibody with high specificity for human Scl. It has been investigated as bone-forming drug among osteoporotic patients with increased risk of fractures. The first clinical study was a phase I randomized, double blind trial conducted in a cohort of healthy men and postmenopausal women (71) The subjects were randomized to receive subcutaneous or intravenous romosozumab or placebo. Administration of romosozumab was accompanied by an increase of serum levels of bone formation markers P1NP, bone-specific alkaline phosphatase (BSAP), osteocalcin, and decreased bone resorption CTX (71) compared with placebo. The study of romosozumab effects on trabecular and cortical bone was assessed in subject with low bone mass in phase I-II studies. The authors observed a significant improvement in vertebral trabecular and cortical bone maintained during the off-treatment follow-up period (72, 73). Moreover, romosozumab was superior to the bisphosphonate aldronate and teriparatide, in increasing bone formation and reducing bone resorption. Romosozumab administration, in phase a III trial, was associated with a lower risk of vertebral and clinical fractures as compared with placebo treatment. A more recent study compared the effectiveness of starting with romosozumab and transitioning to antiresorptive agent alendronate vs. alendronate alone in reducing the risk of fracture among postmenopausal women with osteoporosis (74). Treatment with romosozumab before aledronate reduced the risks of a new vertebral, clinical, nonvertebral, and hip fracture compared to alendronate alone associated with a rapid gain in BMD.

Blosozumab (Eli Lilly and Company, Indianapolis, IN, USA) is a humanized monoclonal IgG4 antibody targeted against Scl that displayed similar bone anabolic properties to romosozumab. Specifically, results of a randomized, placebo-controlled phase II clinical trial in postmenopausal women with low BMD demonstrated that blosozumab increased bone formation and spine and total hip BMD, while decreasing bone resorption (10).

The significant decrease in biochemical markers of bone resorption observed with both drugs may be related to a decreased RANKL and increased OPG levels, with a reduction in the RANKL/OPG ratio and in bone resorption.

BPS804 (Novartis, Basel, Switzeland) is a human IgG2 Scl-Ab being evaluated in clinical trials for osteogenesis imperfecta (OI) has demonstrated a stimulatory effect on bone formation and inhibitory effect on bone resorption (75).

Some limitation for the use of both drugs, came from the studies reporting a reduction of circulating bone formation and resorption markers to baseline levels within a year (10, 73). This effect may be partly due to a Scl-independent bone response: the reduced stresses and strains within the skeleton following the new bone formation, determines a reduction of positive signal for bone formation (76). In addition, Dkk-1, which is upregulated in Scl deficiency (77) might reduce bone formation as a compensatory mechanism in the absence of Scl. Moreover, these Scl-Abs showed immunogenic properties leading to the development of anti-drug antibody (ADA) even after short-term treatment (71, 75). However, this phenomenon does not affect the pharmacodynamics and pharmacokinetic properties and does not induce adverse effects. Both pre-clinical and clinical data showed that Scl-Ab administration increased the expression of SOST and the level of serum Scl that decreased after discontinuation. These effects might be due to either the formation of Ab-Scl complex or the presence of a feedback mechanism by which the blockade of Scl triggers its production (67, 78).

## Myeloma bone disease

Bone remodeling alteration is one of the hallmarks of MM (79). In this hematological malignancy, the plasma cell accumulation into the BM leads to bone destruction due to a severe unbalanced and uncoupled bone remodeling (80, 81) Indeed an increase of OCL enrollment and activity together with a deep OB suppression have been shown in MM patients (80, 81). MM bone disease occurs in about 80% of MM patients at diagnosis (82), resulting in pathological fractures, spinal cord compression and pain, significantly impacting their quality of life (80, 81). BPs, such as zoledronic acid and pamidronate, are the current treatments of choice for MM bone disease. BPs bind avidly to bone matrix and are incorporated into areas of active bone remodeling (83). During bone resorption OCLs incorporate BPs, leading to reduced OCL recruitment, maturation and activity (83).

Either soluble factors or the cell-to-cell contacts between MM and microenvironment cells are involved in bone alterations, resulting in the stimulation of OCL formation and activity, and inhibition of OB differentiation. These alterations of BM microenvironment and, consequently MM bone disease development, provide a permissive niche that promotes growth and survival of MM cells (80, 81). Several cytokines and chemokines contribute to the bone remodeling alterations in MM. These soluble factors are directly released by MM cells and/or produced by stromal and osteoprogenitor cells after interaction with MM cells. Indeed, the cell-to-cell interaction with MM cells, upregulates RANKL while downregulates OPG production in stromal cells, sustaining OCL recruitment and survival (80, 81). Furthermore chemokine (C-C motif) ligand (CCL)-3, interleukin (IL)-1, IL-3, IL-6, activin A, and tumor necrosis factor (TNF) α are known to be upregulated into the MM BM microenvironment and involved in OCL formation (80, 81, 84–86).

The interaction between MM cells and stromal cells also inhibits in stromal cells the activity of Runx2, the main pro-osteoblastogenic transcription factor, leading to the suppression of OB differentiation (87). Moreover, MM patients show high BM levels of cytokines such as IL-7 and HGF that contribute to the Runx2 inhibition and osteoblastogenesis decrease (88, 89). Together with their role in MM-induced enhanced osteoclastogenesis, IL-3 and Activin A also have a role in OB inhibition in MM patients (90, 91). Lastly, it has been shown that MM patients have high BM level of several Wnt signaling inhibitors such as Dkk-1, soluble frizzled related protein (sFRP)-2, and sFRP-3, that contribute to MM-induced OB suppression and MM bone disease (80, 88, 92–94).

## Osteocyte and myeloma bone disease

As described above, bone destruction in MM relies upon the exchange of soluble factors as well as the interactions between MM cells and OCLs and OBs. Nevertheless, little is known about the interplay between MM cells and osteocytes and their role in MM bone disease. A preliminary paper by Eisenberger et al. (95) presented a transcriptome analysis of the *in vivo* effects of MM cells on osteocytes. The study clearly demonstrated that MM-induced stress generated specific gene expression footprints in osteocytes. More recently, a histological study performed on human bone biopsies, revealed that MM patients were characterized by increased osteocyte death and fewer viable osteocytes when compared with healthy controls (3). Moreover, the presence of osteolysis in MM patients correlated with the increased osteocyte death, probably due to increased osteocyte apoptosis. Interestingly, MM patients, when compared to healthy controls or monoclonal gammopathy of undetermined significance (MGUS) patients, showed a higher number of OCLs negatively correlating with the number of viable osteocytes. The same study showed that in a co-culture system, MM cells upregulated the production of pro-osteoclastogenic molecules such as IL-11, Matrix metalloproteinase-1 (MMP-1), and CCL3/macrophage inflammatory proteins (MIP)-1α by preosteocytes (3). Indeed, the conditioned media of these co-cultures increased the *in vitro* OCL formation that was inhibited by the presence of anti-CCL3 and anti-IL11 antibodies. The immunohistochemical analysis of bone biopsies showed that the osteocytic expression of IL-11 was higher in osteolytic MM patients when compared to non-osteolytic ones, even though there were no differences between MM and MGUS patients. Later, the same group demonstrated that MM cells induced autophagic cell death in co-cultured osteocytes, thus supporting the notion that other mechanisms, other than apoptosis, underlie the role of osteocytes in MM bone disease (2).

Osteocytes are in direct contact with MM cells in MM-bearing mice and so, these interactions increase apoptosis and the production of RANKL and Scl by osteocytes (5). *In vitro* experiments demonstrated that the activation of Notch signaling underlined the increased osteocytic apoptosis resulting in: (1) increased expression of RANKL and ability of osteocytes to recruit OCL precursors, and (2) increased production of Scl, which in turn inhibits Wnt signaling and OB differentiation. No less important, this physical interaction induces the reciprocal activation of Notch pathway in osteocytes and MM cells, supporting the growth, and proliferation of tumor cells (5). One of the possible MM-factors responsible for increased osteocyte death was TNF-α, as recombinant TNF-α increased osteocyte apoptosis and neutralizing anti-human TNFα antibody blocked the MM-induced reduction of osteocyte viability (5).

**Figure 2:**
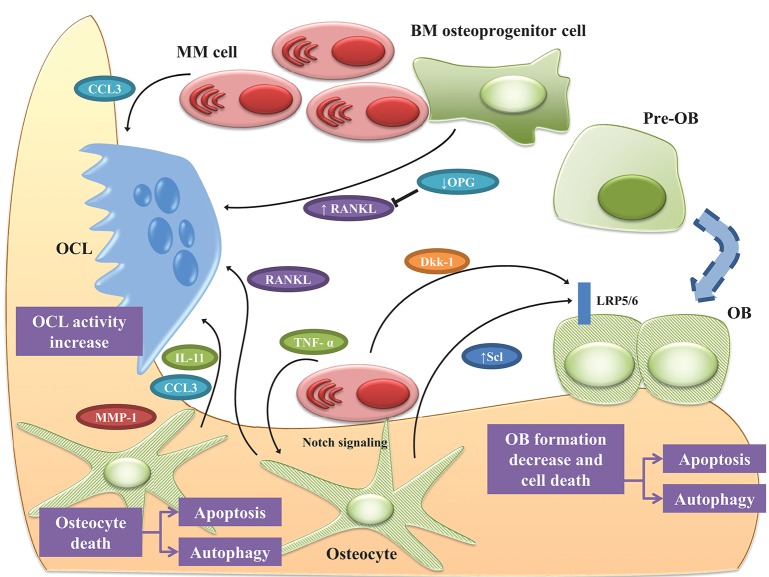
Osteocyte role in MM bone disease. Bone destruction in MM rely up the exchange of soluble factors as well as the interactions between MM cells and OCLs and OBs. Osteocytes play a pivotal role in orchestrating this interplay. Cell-to cell interaction with MM cells, upregulates RANKL while downregulates OPG in osteoprogenitor cells, thus stimulating OCL survival. Under MM stimuli, osteocytes and OBs undergo apoptosis and autophagic cell death. In this scenario, osteocytes produce the pro-osteoclastogenic factors IL-11, CCL3, and MMP1 increasing OCL activity. The physical contact between MM cells and osteocytes induce the reciprocal activation of Notch pathway resulting in increased expression of RANKL, which stimulates OCL, and Scl, which suppress bone formation by osteocytes as well as MM cells growth and osteocyte apoptosis. TNF-α produced by MM cells exacerbated these effects. The effects of MM cells on osteocytic expression of Scl is controversial since some authors reported that osteocytes isolated from tumor-bearing mice expressed lower Scl than non-tumor bearing mice. Moreover, MM cells induce the expression of Scl in OBs via secretion of Dkk-1. See text for details.

Together these data suggest that, in MM-colonized bone, osteocytes are responsible for the increased OCL recruitment as well as the inhibition of bone formation through cell-to-cell interactions and release of soluble factors. The complex interplay between MM cells and osteocytes is shown in Figure 2.

## Osteocyte as therapeutic targets in MM

The recent improvements in the knowledge of osteocyte role in MM bone disease, have raised the possibility of targeting osteocytes as new therapeutic strategy to treat bone disease. Different studies sought to determine the effects of the main anti-MM drugs, such as proteasome inhibitors (PIs), as well as anti-resorptive agents BPs, and PTH on osteocytes. The first observation came from a study by Terpos et al. reporting a reduction of serum levels of Scl in MM patients receiving four cycles of bortezomib monotherapy. On the basis of this evidence, Toscani et al. investigated the effect of bortezomib therapy on osteocyte viability on BM biopsies taken from MM patients. Interestingly, patients treated with a bortezomib-based regimen showed a significant higher number of viable osteocytes compared with those treated without bortezomib. Additionally, bortezomib counterbalanced the negative effect of dexamethasone on osteocyte viability. A similar reduction of apoptotic osteocytes was also observed (2). In keeping with data described above reporting the ability of MM cells to induce autophagic cells death in cocultured osteocytes, *ex vivo* analysis showed that patients treated with bortezomib had a reduction of autophagic osteocytes compared with controls treated without bortezomib thus confirming the great impact of proteasome inhibition in preventing osteocyte death. *In vitro*, PIs were also able to block osteocyte death induced by MM cells and high doses of dexamethasone by inhibiting the activation of the autophagic pathway and the formation of autophagosome (2). Also, BPs are able to target osteocytes. It has been reported that osteoporotic patients treated with BPs had increased levels of serum Scl and reduced bone turnover markers (96). The mechanism by which BPs might modulate Scl levels remains unclear. It has been suggested that BPs induce the accumulation of Scl-secreting OCL precursors (96). Others linked the effect of BPs on Scl levels to the anabolic effects of intermittent PTH (97, 98).

## Preclinical studies with anti-scl antibody in MM

Several clinical studies showed that patients with active MM were characterized by high levels of circulating Scl, which correlated with the presence of osteolytic fractures, disease stage and biochemical markers of bone remodeling (7, 99). There are controversial reports regarding the cellular origin of Scl in MM. Some authors showed that MM cells directly produced Scl (100) or were able to induce its production by osteocytes (5, 6). Nevertheless, Giuliani et al. did not find any significant difference in the expression of Scl in bone biopsies of MM patients (3).

More recently, Eda et al. identified spindle-shaped BM stromal cells and OBs as the main source of Scl in BM biopsy samples from MM patients (101), suggesting that, other than osteocytes, these cells are responsible for the increased levels of Scl in MM patients.

Delgado-Calle et al. generated a MM immunodeficient mouse model with a global deletion of SOST (Sost–/–) injected with MM cells. Interestingly, the mice displayed decreased osteolysis and improved bone loss compared with wild type mice, without affecting MM growth (8). Moreover, whereas MM-injected wt mice displayed reduced bone surface and OB number, MM-injected Sost–/– mice did not display a reduction in the number or function of OBs suggesting that Scl is involved in the OB suppression induced by MM cells.

For further insight into the cellular effects of Scl inhibition, the authors treated an established MM immune-competent mouse model with Scl-Ab.

Mice receiving Scl-Ab showed reduced osteolysis and increased bone formation compared with mice receiving control IgG, no differences in MM growth and with a modest effect on OCLs (8). Furthermore, the increased bone volume was present in mice with both low and high tumor burden suggesting that the anabolic effect is independent of tumor cells presence.

By using a human MM xenograft mouse model, Eda et al. showed that, compared to controls, MM-bearing mice presented high levels of mouse Scl, together with the inhibition of activated β-catenin expression in bone (101).

The treatment with Scl-Ab determined an increase of bone volume and bone formation markers osteocalcin and P1NP as well as the increase of β-catenin staining in xenograft mouse bones. Interestingly, the combination therapy with carfilzomib increased bone formation together with important reduction of tumor burden when compared with mice treated with carfilzomib alone. Moreover, MM cells induced the expression of SOST in cocultured mature human OBs, via secretion of Dkk-1, and the treatment with neutralizing Scl-Ab blocked MM-induced OB suppression. Importantly, neutralizing Dkk-1 antibody blocked SOST upregulation induced by MM while recombinant Dkk-1 increased SOST expression in immature and mature OBs (101). RNA-seq analysis performed on osteocytes isolated from non-tumor bearing mice revealed that these cells expressed *Sost, Dkk1* and other Wnt antagonist such as *Sfrp1, Sfrp2* and frizzled-b (*Frzb*) (6). In contrast with previous results, the expression of *Sost* and *Dkk1* decreased in osteocytes isolated from tumor–bearing mice compared to naive non-tumor–bearing mice. This suggests that osteocytes respond differently in presence of MM cells although further studies are needed to clarify this aspect.

Given the data demonstrating that Dkk-1 is a direct transcriptional target of β-catenin (102), Florio et el. measured Dkk-1 expression in whole-bone lysate in SOST knockout mice and mice treated with Scl-Ab. Dkk-1 was found significantly upregulated after Scl-Ab treatment probabily due to a negative feedback regulation to prevent excessive bone accrual (70).

A bispecific antibody against Scl and Dkk-1 has been developed recently. In rat, mice and primates, the treatment increased bone mass and bone strength, and improved fracture repair while decreasing bone resorption. These effects were associated with a consistent upregulation of osteoblastic genes *Dkk1, Bglap, Opg*, and *Runx2* and osteocyte activity markers *SOST* and *MEPE* (70). Furthermore, treatment with a bispecific antibody induced a compensatory increase in other secreted Wnt antagonists such as WIF1 and SFRP4, thus suggesting a feedback regulation.

In view of a more realistic therapeutic strategy combining Scl-Ab and available anti-MM drugs, several groups are spending resources in this field. The *in vitro* treatment of MM cells with Scl-Ab in combination with anti-MM drugs, such as bortezomib and dexamethasone, did not affect their anti-MM activity thus promoting the use of combination therapy to improve bone disease and inhibit tumor progression (101). Lastly, a combination therapy of Scl-Ab and zoledronic acid and other anti-MM drugs has been tested. Delgado-Calle et al. demonstrated that Scl-Ab therapy did not impact negatively the anti-MM efficacy of Bortezomib and Dexamethasone *in vitro* (8), while others reported a superior effect of Scl-Ab combined with Zoledronic Acid in increasing bone volume and resistance to fracture *in vivo* (6). This data emphasizes (i) the importance of targeting Scl to improve bone disease in patients with skeletal disorders, (ii) the efficacy of therapies combining Scl-Ab and anti-MM drugs and antiresorptive agents, (iii) the feasibility of evaluating combinatory treatment in clinical studies in patients with MM.

## Conclusions

MM patients'quality of life is strongly affected by the high incidence of bone pain, fractures and other skeletal-related events. Currently, few therapies are approved for the treatment of MM bone disease strongly encouraging the identification of new therapeutic approaches. Together with the physiological role of osteocytes in bone remodeling, recent studies highlight the involvement of osteocyte-MM cell interaction in the pathogenesis of MM bone disease. Numerous reports demonstrated that Scl, an inhibitor of canonical Wnt pathway, is a negative regulator of bone formation and plays a pivotal role in MM bone alterations thus supporting the use of anti-Scl therapy for the treatment of skeletal disease. Scl-Abs have been recently developed showing a good bone anabolic response in osteoporotic patients. Nevertheless, this anabolic effect is transient and followed by anti-catabolic effect with a net increase in bone mass. So far, there are no clinical trials in MM patients but numerous preclinical models of MM demonstrated that the use of Scl-Ab stimulated bone formation. Some concerns came from the controversial observations on the modulation of osteoclastogenic factors as well as increased levels of other Wnt antagonists that counterbalance the inhibition of Scl.

Moreover, since Scl-Ab induced strong bone anabolic responses, it is possible that, prolonged stimulation of bone formation, might cause bony overgrowth and skeletal complications.

Since the levels of Scl change in different diseases and with age, an antibody dose titration might be required. Moreover, the relevance of the increased levels of Scl after Scl-Ab treatment need to be clarified especially considering that other cell types, beyond osteocytes, produce Scl. It is conceivable that, upon inhibition of Scl, other cells are stimulated to produce Scl as a feedback mechanism. Lastly, the effects of ADA on the efficacy of drugs in patients treated with Scl-Ab therapy should be considered. To conclude, the immunotherapy approach targeting Scl appears to be promising also for the treatment of MM bone disease.

## Author contributions

DT, MF, CP wrote the manuscript. NG and MB organized and edited the manuscript.

### Conflict of interest statement

The authors declare that the research was conducted in the absence of any commercial or financial relationships that could be construed as a potential conflict of interest.
